# Redox Biomarker Baseline Levels in Cattle Tissues and Their Relationships with Meat Quality

**DOI:** 10.3390/antiox10060958

**Published:** 2021-06-15

**Authors:** Zoi Skaperda, Angeliki Argyriadou, Paraskevi Maria Nechalioti, Maria Alvanou, Sotiria Makri, Efterpi Bouroutzika, Ioannis D. Kyriazis, Fotios Tekos, Aristidis S. Veskoukis, Theodoros Kallitsis, Robin Mesnage, Georgios Arsenos, Demetrios Kouretas

**Affiliations:** 1 Department of Biochemistry and Biotechnology, University of Thessaly, Viopolis, Mezourlo, 41500 Larissa, Greece; zoskaper@bio.uth.gr (Z.S.); pnechalioti@bio.uth.gr (P.M.N.); maalvanou@uth.gr (M.A.); somak@uth.gr (S.M.); ioankyriazis@uth.gr (I.D.K.); ftekos@uth.gr (F.T.); veskoukis@uth.gr (A.S.V.); 2 Laboratory of Animal Husbandry, School of Veterinary Medicine, Faculty of Health Sciences, Aristotle University of Thessaloniki, 54124 Thessaloniki, Greece; argyrian@vet.auth.gr (A.A.); tgkallitsis@vet.auth.gr (T.K.); arsenosg@vet.auth.gr (G.A.); 3 Veterinary Faculty, University of Thessaly, 43100 Karditsa, Greece; bouroutz@vet.uth.gr; 4 Department of Nutrition and Dietetics, University of Thessaly, Argonafton 1, 42132 Trikala, Greece; 5 Gene Expression and Therapy Group, King’s College London, Faculty of Life Sciences & Medicine, Department of Medical and Molecular Genetics, 8th Floor, Tower Wing, Guy’s Hospital, Great Maze Pond, London SE1 9RT, UK; robin.mesnage@kcl.ac.uk

**Keywords:** antioxidants, redox biomarkers, cattle, meat quality

## Abstract

Cattle breeds or crossbreds with high productivity traits have been developed to meet a growing demand for food. When intensive farming practices are followed, animals face several challenges which can result in poor performance, compromised welfare and the reduced quality of their products. Our study aims to highlight the resting values of the physiological oxidative stress that three cattle breeds exhibit, and their potential relationship with meat quality. For this purpose, we determined the levels of five common redox biomarkers (glutathione (GSH), catalase (CAT), total antioxidant capacity (TAC), thiobarbituric reactive substances (TBARS) and protein carbonyls (CARBS)) in the tissues of three commonly used beef cattle breeds (Charolais (CHA), Limousin (LIM) and Simmental (SIM)) and their association with specific meat quality traits that depend on color, pH and texture. The results revealed that LIM cattle breed animals have elevated intrinsic antioxidant defense systems in comparison to CHA and SIM cattle breed animals. In addition, the meat quality parameters were associated with the redox biomarkers. We propose that the determination of specific antioxidant parameters in the blood might be used as potential biomarkers to predict meat quality. This would allow farmers to nutritionally intervene to improve the quality of their products.

## 1. Introduction

The world population is expected to exceed 9 billion by 2050, with the majority of people living in urban environments. The need for food is predicted to escalate, exceeding the present needs by up to 70% [1]. An increased demand for highly nutritional protein-rich foodstuffs like animal products is expected. Global animal-derived food production is highly dependent on livestock, mainly ruminants, which adapt to a wide-range of ecosystems [2]. They hold a dominant position in the production of meat products [3]. Ruminants can be reared in conditions ranging from harsh climate conditions with limited vegetation in the form of pasture to fully intensive systems intended to maximize productivity [4]. As a consequence, ruminants have been subjected to breeding and genetic selection in order to improve traits with financial importance and keep up with the specific needs of different rearing conditions [2]. Sheep, goat and cattle breeds with enhanced productive orientations (meat or dairy) are available nowadays. In the case of cattle, specific dairy (e.g., Holstein–Fresian) or beef (e.g., Limousin, LIM; Charolais, CHA; Simmental, SIM) breeds are so productive that they have become the dominant rearing breeds, replacing indigenous breeds in many countries. Hence, specific breeds or crossbreds with high productivity traits have been developed.

The productivity of these breeds and the quality of their products are continuously challenged due to altered environmental conditions, intensive farming practices and diseases that affect the animals’ health and well-being [5,6]. Diseases can impact animal productivity and meat quality, with subsequent financial consequences and human health impacts. This has been demonstrated by a meta-analysis linking animal diseases with a decrease of the livestock population in low-income countries by 18% [7]. Moreover, cattle breeding selected solely because of their enhanced productivity allows several diseases to emerge (dairy cows mastitis, beef cattle respiratory disease), ultimately compromising the overall farming system performance as this neglects adaptability traits that these cattle breeds possess to cope with environmental changes [8]. In this context, disease outbreaks in farm animals are facing an unprecedented increase, and therefore possible animal welfare and productivity issues. As a consequence, concerns about the final meat product quality have gained research attention.

Malnutrition, inadequate husbandry practices and diseases are all stimuli that promote the overall stress in the animal physiology mediated by an increased cellular reactive oxygen species [9]. A direct consequence is the oxidation/oxidative modification of meat, which represents a key factor responsible for meat quality degradation, i.e., flavor, color, texture and nutritive value [10]. Considering that meat’s oxidative stability depends on the balance between anti- and pro-oxidant substrates [11], the redox state of farm animals could be an important factor determining their meat quality. The excessive generation of reactive oxygen species is known to lead to lipid peroxidation [12] and protein oxidation [13], which constitute the main non-microbial causes of quality deterioration in meat products [14]. It is crucial to determine how the endogenous antioxidant protective systems which consist of small peptides such as glutathione, carnosine and several other molecules (vitamin E, vitamin A) contribute to the total antioxidant activity levels [15,16]. Some of the antioxidant defensive systems are also antioxidant enzymes, namely superoxide dismutase, catalase and glutathione peroxidase, which protect cells from oxidative damage in vivo [17]. These antioxidant enzymes also ensure stability during post-mortem processes, such as distribution and refrigeration storage [18]. Hence, they can offer, even post-mortem, a level of protection against meat oxidative modifications induced by reactive species [19].

Several studies have examined the use of low-cost antioxidant nutritional interventions in farm animals [20,21]. These studies also determined the redox-related parameters in tissues, and interestingly reported improved animal welfare. However, a significant number of studies showed contradictory results [22,23,24,25,26,27,28]. Antioxidant treatments with vegetable oils, vitamins and polyphenolic extracts in monogastric and ruminant species failed to improve the redox profile in these studies. This discrepancy may be attributed to the suppression of the endogenous antioxidant system, the use of sub-effective treatment doses, or the limited bioavailability of the treatments used.

Consequently, the effects of antioxidant supplementation on livestock health, performance and product quality have been inconsistent. Additionally, it has recently become evident that the contribution of each antioxidant, when measured separately, does not reflect the antioxidant status of meat [29]. Thereafter, the estimation of the overall antioxidant status could be beneficial in describing the capacity of tissues to resist oxidation processes. Moreover, re-designing interventions to improve the animal product quality requires the initial determination of the antioxidant biomarker basal levels. A first aim of this study was to determine the baseline levels of five widely used redox biomarkers (the reduced form of glutathione, catalase, total antioxidant activity, thiobarbituric acid reactive substances and protein carbonyls) in five tissues (blood, liver, diaphragm, psoas major and quadriceps muscles) of three different cattle breeds reared in Greece (namely CHA, LIM and SIM). We then assessed the consequences on the meat quality via colorimetry, pH measurement and texture profile analysis. To our knowledge, this is the first study performed to assess the resting values of redox biomarkers with translational potential in cattle and their relationship with meat quality.

## 2. Materials and Methods

### 2.1. Animals

Thirty-four (34) bulls, aged 19.8 ± 3.8 months, were used for this study. They were either purebred or crosses of 3 commonly used beef cattle breeds (i.e., LIM (*n* = 11), CHA (*n* = 13), and SIM (*n* = 10)). The classification of the animals was performed according to the method approved by the EU age metric system (A–E) (1026/91/EU), and they were rated as A (non-castrated bulls under 2 years old). Because the use of steroid implants is illegal in EU region, all of the animals were steroid free. Purebreds of each breed were determined based on their passport information. In the cases of bulls that were indicated as beef cattle crossbreds according to their passports, the allocation to one of the 3 breeds was based on their phenotypic characteristics that were similar to those of purebreds. Briefly, all of the animals received water *ad libitum* and the same diet, which included the ingredients presented in the Supplementary Table S1. There was a pre-determined threshold of 650 kg body weight for the animals to be considered subject to slaughtering. All of the animals were imported to Greece from other European Union (EU) countries and raised in one farm for a minimum of five months before slaughter. The slaughtering took place in an approved commercial facility, according to EU standards (854/2004). The age at slaughter, hot carcass weight and carcass conformation were recorded. The age at slaughter (months) was calculated based on the culling date and the date of birth of each bull, as stated in their passports. The hot carcass weight (kg) was determined at the end of the slaughter line, after the completion of the carcass preparation and immediately prior to refrigeration. The carcass conformation was classified based on the SEUROP scale [30] by the official state veterinarian.

### 2.2. Sample Collection

Blood samples from the jugular vein were collected in blood collection tubes (EDTA, BD Vacutainer^®^ Blood collection tubes, BD, USA) immediately after slaughter. The tissue collection was also performed on the same day as the blood sampling, immediately after the tissues were accessible. Samples of the liver and muscle (i.e., psoas major, quadriceps and diaphragm) were excised (10 g per sample) and kept in plastic cassettes. Briefly, the diaphragm muscle was derived from the *pars costalis* due to its easy and rapid accessibility. Concerning the quadriceps muscle, we sampled the *rectus femoris* muscle, especially the edge near the end of the femur. The *psoas major* was collected from the muscle part that, together with the gluteus muscle, inserts on the trochanter minor. We collected the part of the liver in which the duodenum is impressed. The samples were immediately placed in liquid nitrogen and transferred within 3 h to be stored at −80 °C until the analyses. Approximately 24 h following slaughtering, a sample of meat 2–3 cm thick was obtained from the 13th rib of the left side of each cold carcass. The meat samples were vacuum-packed and stored at 4 °C for six days until they were used for the assessment of the meat quality.

### 2.3. Blood Fractionation

The blood samples were centrifuged immediately after collection at 1370× *g* for 10 min at 4 °C. Afterwards, the plasma was collected and used to measure the TAC, TBARS and CARBS levels. The packed erythrocytes were lysed with distilled water (1:1 *v/v*), inverted vigorously, centrifuged at 4000× *g* for 15 min at 4 °C, and the erythrocyte lysate (RBCL) was collected for the measurement of the GSH and catalase activity. Regarding the measurements of the GSH concentration, 400 μL erythrocyte lysate were added to 400 μL 5% trichloroacetic acid (TCA), inverted vigorously, centrifuged at 15,000× *g* for 5 min at 4 °C, and the supernatant was collected. Then, 90 μL 5% TCA was added to each tube, inverted again vigorously, centrifuged as previously and, finally, the clear supernatant was collected.

The hemoglobin concentrations were determined by the hemiglobincyanide (HiCN) method using a commercial kit (Dutch Diagnostics, Zutphen, Holland). Briefly, 5 μL RBCL was added to 1 mL working hemoglobin reagent (reagent R1). The reagent R1 (pH 7.3) consisted of potassium hexacyano ferrate (III) (0.607 mmol/L), potassium cyanide (0.767 mmol/L), potassium dihydrogen phosphate (1.03 mmol/L) and 0.05% detergent. The samples were vortexed and incubated in a dark place for 10 min, and then the absorbance was measured at 540 nm. In each experiment, 1 mL R1 was used as a blank.

### 2.4. Tissue Homogenization and the Measurement of the Total Protein Concentration

The tissue homogenization was performed using a Bertin Technologies homogenizer. In brief, 200–250 mg of each sample and 600–750 μL PBS with diluted protease inhibitors (CompleteTM mini protease inhibitors) were transferred into homogenization tubes and the samples were homogenized for 30 s at the highest available speed. Then, the homogenate was centrifuged (15,000× *g*, 5 min, 4 °C) and the supernatant crude protein extract was collected in new Eppendorf tubes. The total protein concentration was determined using the Bradford method, and the samples were stored at −80 °C until the analysis.

### 2.5. Redox Biomarkers Determination

Each assay was performed in triplicate and within 3 months of the blood collection. The blood samples were aliquoted at −80 °C and thawed once before the analysis.

#### 2.5.1. Reduced form of Glutathione (GSH)

The GSH concentration was measured according to a slightly modified version of the method of Reddy et al. [31], as previously described by Veskoukis et al. [32]. Briefly, 20 μL RBCL or 100 μL tissue homogenate was mixed with 5% trichloroacetic acid (TCA), the samples were centrifuged (15,000× *g*, 5 min, 5 °C) and then the supernatant was transferred to a new Eppendorf tube. After that, 20 μL TCA-treated tissue homogenate was mixed with 660 μL sodium potassium phosphate buffer (67 mM, pH 8) and 330 μL 5,5′-dithiobis-2 nitrobenzoate (DTNB; 1 mM). The samples were incubated in the dark at room temperature for 15 min and the optical density was measured at 412 nm.

#### 2.5.2. Catalase (CAT)

The catalase (CAT) activity was determined based on a slightly modified version of the method of Aebi [33], as previously described by Veskoukis et al. [32]. Specifically, 4 μL erythrocyte lysate (diluted 1:10) or 5 μL liver homogenate (diluted 1:5) οr diaphragm, pelvis and quadriceps, undiluted, was added to 2991 or 2990 μL of 67 mM sodium potassium phosphate buffer (pH 7.4), respectively. Subsequently, the samples were incubated at 37 °C for 10 min. In total, 5 μL 30% H_2_O_2_ was added to the samples and the change in absorbance was immediately read at 240 nm for 130 sec. The CAT activity was calculated based on the molar extinction coefficient of H_2_O_2_ (43.6 M^−1^ cm^−1^).

#### 2.5.3. Total Antioxidant Activity (TAC)

The determination of the TAC was based on the method of Janaszewska and Bartosz [34]. The reaction was performed in the dark at room temperature for 60 min in a final volume of 1 mL that contained 40 μL tissue homogenate or 20 μL plasma, 460 or 480 μL phosphate buffer (10 mM; pH 7.4), respectively, and 500 μL 2,2-diphenyl-1-picrylhydrazyl radical (DPPH, 0.1 mM) solution. The samples were centrifuged (15,000× *g*, 3 min) and the optical density was read at 520 nm.

#### 2.5.4. Thiobarbituric Acid Reactive Substances (TBARS)

For the TBARS, a slightly modified version of the assay of Keles et al. [35] was used as previously described by Spanidis et al. [36]. More elaborately, 100 μL plasma or tissue homogenate was mixed with 500 μL 35% TCA and 500 μL Tris–HCl (pH 7.4). The samples were incubated for 10 min at room temperature. Then, 1 mL containing Na_2_SO_4_ (2M) and thiobarbituric acid (TBA; 55 mM) was added and the samples were incubated for 45 min at 95 °C. Afterwards, the samples were transferred at 4 °C for 5 min, followed by the addition of 1 mL 70% TCA and homogenization using a vortex. From each sample, 1 mL was transferred to Eppendorf tubes and the samples were centrifuged (11,200× *g*, 3 min). Finally, 900 μL of the supernatant was transferred into a plastic cuvette and the optical density was determined at 530 nm. The calculation of the TBARS concentration was based on the molar extinction coefficient of malondialdehyde (155 × 10^3^ M^−1^cm^−1^).

#### 2.5.5. Protein Carbonyls

The protein carbonyl determination was based on a slightly modified version of the method of Patsoukis et al. [37], as previously described by Veskoukis et al. [32]. Briefly, 50 μL 20% TCA was added to 50 μL plasma or tissue homogenate, and the mixture was incubated for 15 min at 4 °C, followed by centrifugation (15,000× *g,* 5 min, 4 °C). Subsequently, the supernatant was discarded and the pellet was resuspended in 500 μL 10 mM 2,4-dinitrophenylhydrazine (DNPH) (diluted in 2.5 N HCl). Every sample had its own blank that constituted of the same sample volume resuspended with 500 μL 2.5 N HCl without DNPH. The samples and their respective blanks were incubated in the dark at room temperature for 1 h with intermittent vortexing every 15 min, followed by centrifugation (15,000× *g*, 5 min, 4 °C). After the centrifugation, the supernatant was discarded and the pellets were resuspended with 1 mL TCA (10%). After the resuspension, the samples and blanks were again centrifuged (15,000× *g*, 5 min, 4 °C) and the pellets were cleaned with 3 washes with 1 mL ethanol–ethyl acetate mixture (1:1 *v/v*). The pellets were resuspended while the samples and blanks were centrifuged each time at 15,000× *g* for 5 min at 4 °C. After the third wash, the pellets were resuspended with 1 mL urea (5 M; pH 2.3) and the samples or blanks were vortexed and incubated at 37 °C for 15 min. The samples and blanks were centrifuged (15,000× *g*, 5 min, 4 °C) and their optical density was determined at 375 nm. The calculation of the protein carbonyl concentration was based on the molar extinction coefficient of DNPH (22 × 10^3^ M^−1^cm^−1^).

### 2.6. Meat Quality Assessment

The texture profile analysis, pH measurement and meat colorimetry were performed on each meat sample from the 13th rib. The meat color values were measured on freshly exposed meat samples immediately after unpacking, using the Konica Minolta CR-410 Chroma-Meter with a 50 mm aperture size, illuminant C and a 2° observer. Prior to scanning, the colorimeter was calibrated with a white tile (Y: 94.8/X: 0.3130/y: 0.3190). With the colorimeter always perpendicular to the myofibrils, each meat sample was scanned three consecutive times at different positions, avoiding fat and connective tissue. The measured values of lightness (L*), redness (a*) and yellowness (b*) were averaged over each sample. The chroma and hue angle values of each sample were calculated based on the a* and b* averages according to the following formulae, as described by [38]:

Chroma (saturation index) = (a*^2^ + b*^2^)^1/2^ (1)

Hue angle = arctangent (b*/a*)(2)

The meat pH was measured with the Mettler Toledo FiveGo pH-meter, non-destructively, after piercing a hole into each sample. Two consecutive measurements were performed on the same point of incision, and their average value was considered for the statistical analysis that was performed. Prior to its use, the instrument was calibrated with two pH buffer solutions (4.00 and 7.00).

The texture profile analysis was performed using a Stable Micro Systems TA.HD *plus* Texture Analyser with a flat-faced cylindrical 1.27 cm diameter probe, connected to a computer equipped with Exponent software (version 6.1.16.0). An oval-shaped piece of the same width and thickness (2–3 cm) was extracted from the center of each sample and used in a double compression cycle test. The probe moved downwards, perpendicular to the myofibrils of the samples, with a pre-test speed of 1.00 mm/s and a test and post-test speed of 5.00 mm/s in order to achieve a 40% deformation in each cycle. The wait time between the two cycles was 2.02 s. For each sample tested, the software produced a force–time plot representing the resistance of the sample to compression against time. Based on the latter plot, the following parameters were calculated [39,40]:

- Hardness 1: The maximum force during the first compression cycle, representing the hardness of the sample experienced during the first bite.
- Hardness 2: The maximum force during the second compression cycle, representing the hardness of the sample experienced during the second bite.
- Cohesiveness: The ratio of the total work in the second compression cycle to the total work in the first, indicating the degree of the sample’s resistance to deformation during the first compression that was retained during the second compression.
- Springiness: The ratio of the sample height in the second compression to the original height detected in the first compression, indicating how well a sample can return to its original form after being compressed and deformed during the first cycle.
- Chewiness: The outcome of the multiplication of Hardness 1 * Cohesiveness * Springiness, indicating the energy needed to chew the sample to the point where it can be swallowed.

### 2.7. Statistical Analysis

The statistical analyses for the evaluation of the redox-related biomarker levels were performed using an ANOVA test for comparisons of multiple groups. The pairwise comparisons were p-values from a Tukey’s post-hoc analysis. This was calculated in R version 4.0.0 with R in-house functions. The figures were created with the packages ggpubr_0.3.0 and ggplot2_3.3.0.

Regarding the second objective of the present study related to the meat quality assessment, the statistical analysis was performed using R programming language (software version 3.5.1). The outliers of the meat pH and texture profile parameters were first removed. A value was considered as an outlier when it was further away than the sum of the mean ± 3×SD. For the same variables (except for springiness), logarithmic transformations (natural log) were performed in order to achieve the normality of the respective distributions. In the preliminary analyses, the carcass traits with statistically significant effects on the meat quality parameters were determined. Specifically, the hot carcass weight, carcass conformation class, age at slaughter and breed were tested. In the case in which two of the latter traits had statistically significant effects on the same meat quality parameter, further analyses including both effects were performed in order to determine which would remain statistically significant.

Linear regression analyses were performed in order to assess the effect of the redox status on the meat quality parameters. The statistically significant effects identified in the preliminary analyses were included in the models, which were of the following general form:

Y_ghi_ = μ + b_1_*RSg + b_2_*CW_h_ + e_ghi_ (3)

where Y is the dependent variable, μ is the overall population mean, RS_g_ is the fixed effect of each of the redox status variables (25 continuous variables, 5 redox biomarkers, i.e., GSH, TAC, CAT, TBARS and protein carbonyls, measured in samples of 5 tissues, i.e., blood, liver, diaphragm, psoas major and quadriceps muscles), b_1_ is the regression coefficient on RS_g_, CW_h_ is the fixed effect of the hot carcass weight, b_2_ is the regression coefficient on CW_h_ and e_ghi_ is the residual error.

Meat hardness 1 and 2 and springiness were analyzed with the above model (3) including the breed effect (a categorical variable with three levels: LIM and its crosses, CHA and its crosses, SIM and its crosses) instead of the hot carcass weight. Accordingly, the same model (3) was used for the meat chewiness analysis after replacing the hot carcass weight effect with the carcass conformation effect (a categorical variable with four levels: SEUROP classification categories U, U+, R, R+). The level of statistical significance was set at *p* = 0.05.

## 3. Results

For the determination of the redox biomarkers’ baseline levels, we examined different tissues based on their ease of sampling, antioxidant background and the different levels of stress due to physiological daily movement. The liver is the main metabolic system of xenobiotic substances performing important physiological functions such as protein synthesis and food digestion [41]. Additionally, high metabolizing functions of hepatocytes make the liver a tissue with elevated oxygen intermediates production [42]. In order to cope with a potential oxidative stress, the liver has an increased antioxidant defense to counteract oxidative stress and maintain redox homeostasis. The selection of blood as a tested tissue was based mainly on its ease of sampling, its correlation with generalized oxidative stress in the body [43] and the amplified oxygen-related biology of erythrocytes (the most abundant cell type in the body) that have developed a reinforced antioxidant defense system [44]. Regarding the muscle tissues, they were chosen because they are all edible parts of animals which undergo different levels of oxidative stress during their lifetime, ranging from high daily motility, like the diaphragm, to less active quadriceps, and almost static, psoas major. The baseline levels of the redox biomarkers in every tissue examined are shown in Table 1. 

### 3.1. Reduced Form of Glutathione

**Figure 1:**
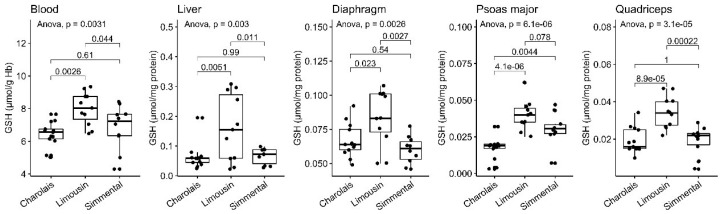
GSH levels in the blood, liver, diaphragm, quadriceps and psoas major of CHA, LIM and SIM cattle.

Reduced glutathione (GSH) represents one of the most abundant intracellular antioxidant non-enzymatic molecules [45], and therefore it is of paramount importance to determine its basal levels in all of the available tissues from the breeds tested. LIM possessed higher basal GSH levels among the studied cattle breeds in all of the tissues examined. The latter observation was statistically significant (*p* < 0.05) in all cases except for the comparison between the LIM and SIM samples of psoas major (*p* = 0.078) (Figure 1, Table 1).

### 3.2. Catalase Activity

**Figure 2:**
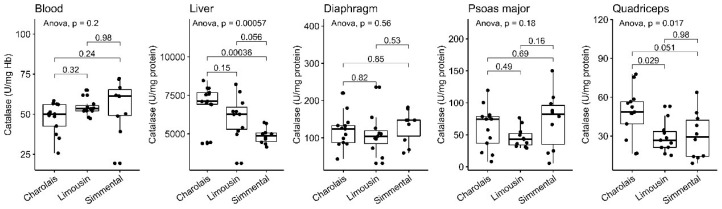
CAT activity in the blood, liver, diaphragm, quadriceps and psoas major of CHA, LIM and SIM cattle.

Catalase (CAT) is an important enzyme which exerts antioxidant activity through the decomposition of hydrogen peroxide into water and molecular oxygen [46]. Therefore, the determination of its activity represents a critical component of the cellular antioxidant defensive network. The differences regarding the CAT activity were not so consistent as in the case of the GSH levels. More specifically, the CAT activity in LIM liver showed a trend of increase when compared to SIM (*p* = 0.056) (Figure 2, Table 1). On the contrary, CHA liver exhibited elevated CAT activity compared to SIM (*p* < 0.001). Finally, the CAT activity was elevated in the quadriceps muscle of CHA cattle compared to LIM (*p* < 0.05), and revealed the same trend when compared with SIM (*p* = 0.051).

### 3.3. Total Antioxidant Capacity Levels

**Figure 3:**
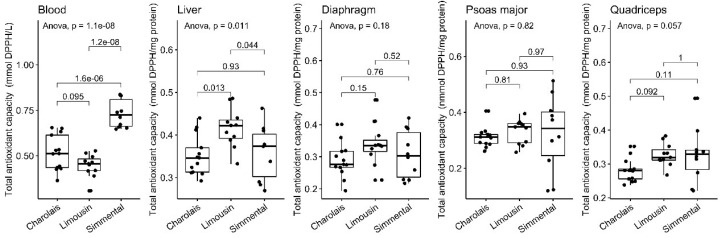
TAC levels in the blood, liver, diaphragm, quadriceps and psoas major of CHA, LIM and SIM cattle.

Subsequently, we determined the total antioxidant capacity (TAC); the major advantage of this test is that it measures the antioxidant capacity of a biological sample as a whole and not just a single antioxidant [47]. TAC was elevated in the liver samples of the LIM breed compared to the other two breeds (*p* < 0.05) (Figure 3, Table 1). On the contrary, the TAC levels were significantly decreased in the blood of the LIM breed compared to the SIM breed (*p* < 0.001). In the same tissue, SIM exhibited the highest CAT levels among the tested breeds (*p* < 0.001).

### 3.4. Lipid Peroxidation Levels

**Figure 4:**
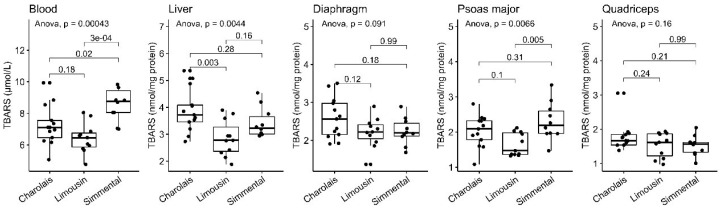
TBARS levels in the blood, liver, diaphragm, quadriceps and psoas major of CHA, LIM and SIM cattle.

The oxidation reactions of lipids, as documented through TBARS levels, are responsible for the reduction of meat’s nutritional value and quality, making this biomarker a crucial factor worth evaluating [12]. Consistent with the enhanced antioxidant efficacy observed, the LIM breed exerted reduced lipid peroxidation in all of the tissues tested apart from the quadriceps and diaphragm in comparison to the other breeds examined (Figure 4, Table 1). More specifically, the LIM breed had lower lipid peroxidation levels compared to the SIM breed in the blood (*p* < 0.001) and psoas major (*p* < 0.01). Similarly, lower TBARS were observed in LIM liver (*p* < 0.01) in comparison to CHA, whereas only statistically non-significant respective trends of reduction were observed in the psoas major.

### 3.5. Protein Carbonyl Content

**Figure 5:**
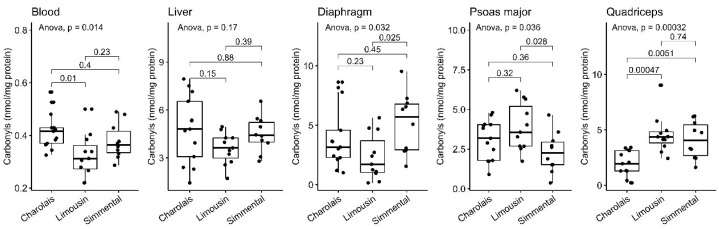
Protein carbonyls levels in the blood, liver, diaphragm, quadriceps and psoas major of CHA, LIM and SIM cattle.

Protein carbonylation is a non-reversable posttranslational modification of proteins as a result of denaturation or proteolysis phenomena, playing a decisive role in meat’s nutritional and quality traits [48]. Therefore, it allowed us to determine the extent to which the level of cellular oxidative stress affected the protein content and quality in the examined tissue. Following the same pattern observed regarding lipid peroxidation, the LIM breed had lower protein carbonylation compared to both the CHA (*p* < 0.01) and SIM breeds in blood; however, the difference compared to the latter breed was not statistically significant. The same trend was observed in the liver; however, the observed differences were not statistically significant (Figure 5, Table 1). The protein carbonylation levels were also reduced in the diaphragm of the LIM breed compared to SIM (*p* < 0.05). By contrast, LIM had elevated protein carbonyls compared to the SIM and CHA breeds in the psoas major (*p* < 0.05) and quadriceps muscle (*p* < 0.001).

### 3.6. Effect of the Redox Status on the Meat Quality Parameters

Descriptive statistics of all of the studied meat quality parameters are presented in Table 2. The statistically significant effects of the redox status on the meat quality parameters are summarized in Table 3. All of the meat color values, except for L*, were significantly affected by blood TAC (*p* < 0.05); specifically, for one unit increase of the latter, the values of a*, b*, chroma and hue angle increased by 4.713, 7.118, 6.285 and 0.239, respectively. The values of a* and chroma were also affected by the TAC levels in the liver (*p* < 0.01); one unit increase of liver TAC decreased a* and chroma by 14.685 and 16.642, respectively. Furthermore, the chroma and b* values decreased by 65.447 and 65.636 (*p* < 0.05), respectively, as a result of a one unit increase in GSH in the quadriceps muscle. The CAT activity in the diaphragm had a very slight effect on the hue angle and b* values (*p* < 0.05); the latter were increased by 0.001 and 0.019, respectively, for a one unit increase in CAT. Lastly, the protein carbonyl activity affected the hue angle and L* values (*p* < 0.05). Specifically, a one unit increase in liver protein carbonyls increased the hue angle by 0.015. L* increased by 0.959 and decreased by 0.559 for a one unit increase in the psoas major and diaphragm protein carbonyls, respectively.

The meat pH was significantly affected by the protein carbonyls in the blood and the GSH in the psoas major muscle. A one unit increase in the latter redox status variables led to lower (by 12.18%, *p* < 0.05) and higher (by 185.32%, *p* < 0.01) meat pH values, respectively.

Regarding the texture profile parameters, meat hardness 1 and 2 were significantly affected by the same redox status variables (*p* < 0.05); a one unit increase in the CAT levels of the blood and psoas major muscle led to higher hardness 1 (by 2.09% and 0.70%, respectively) and hardness 2 values (by 1.96% and 0.67%, respectively), whereas the same increase in the psoas major protein carbonyls levels decreased hardness 1 and 2 by 14.97% and 14.65%, respectively. Accordingly, a one unit increase in the protein carbonyl levels in the diaphragm decreased the chewiness by 7.56% (*p* < 0.05). Increased levels of GSH in the blood were associated with decreased cohesiveness (by 3.02% for a one unit increase of GSH, *p* < 0.05), whereas the respective levels in the quadriceps muscle led to higher springiness (by 5.814 for a one unit increase of GSH, *p* < 0.01).

## 4. Discussion

This study examined beef cattle’s redox status and its effect on meat quality parameters without any nutritional intervention. The results indicate that the LIM cattle breed had a potent antioxidant defense system. Moreover, the meat quality parameters correlated with the redox biomarkers, revealing the potential of using specific biomarkers in animals for the prediction of meat product’s quality.

It is known that the lack of resting values of the redox status parameters in livestock animals is a critical limitation for in vivo nutritional antioxidant treatments that target increased productivity. Therefore, we aimed to measure the redox biomarker baseline values of beef cattle raised in Greece, and to associate them with meat quality parameters. A strong point of the present study is that all of the animals were under the same nutritional management and rearing conditions, and the tissue samples were taken from the same anatomical sites. This approach allowed us to eliminate limitations that usually arise concerning the diverse diets fed to experimental animals [49,50,51]. Hence, the phenotypic sources of variation were eliminated, enabling an objective comparison of the biological parameters of interest.

Productivity is a key word for the livestock industry. Several conditions, such as malnutrition, diseases and problematic husbandry systems have been documented to affect animal health, incriminating oxidative stress as one of the driving forces behind reduced productivity [9,52]. This led producers to treat their animals with antioxidants, hoping that productivity would be boosted. Although the idea conceptually was structured on a scientific basis, dietary intervention with antioxidant supplementation led to contradictory results. Several studies reported that uncontrolled antioxidant treatments negatively affect animal welfare, acting as prooxidant agents [26,27,28]. Animals exposed to oxidative stress react with the compensatory induction of the endogenous antioxidant mechanisms. The effect of nutritional antioxidants, particularly vitamins like carotenoids or flavonoids, on the antioxidant enzymes expression levels is controversial [53]. Additionally, the results of another study introduced the possibility of disease risk related to high vitamin E supplementation without knowing the current redox biomarkers levels in each individual cow [28]. Although these studies examined the redox state biomarkers in different farm animal species, they showed that diets rich in PUFAs using extracts from rosemary, green tea, grape seed and tomato fed to growing birds had no effect on the plasma oxidative status and lipid oxidation [23]. In line with the previous study, high doses of vitamin E in turkeys were not able to alter the activities of several antioxidant enzymes [24]. All of the above could be associated with low bio-availability due to limited absorption or the high rate at which the antioxidants are metabolized [54]. Moreover, it is of paramount importance to consider that the antioxidant supplementation will not allow the defense system to reach a specific threshold due to initially reduced basal levels.

As our results have indicated, although all of the breeds had the same housing and rearing conditions, the LIM breed had elevated basal levels of intrinsic GSH concentration, CAT activity and TAC levels. The improved antioxidant profile of LIM might explain the decreased levels of lipid and protein oxidation that this breed presented in comparison to the CHA and SIM breeds. In line with the present results, it was recorded that the lipid oxidation biomarkers were significantly lower in the LIM breed compared to SIM and three other cattle breeds in China [51]. Enhanced lipid peroxidation levels have also been reported in infected [55,56,57], ketotic [58] or periparturient [59,60] dairy cows, indicating the significant one-dimensional effect that environmental and physiological conditions have on redox parameters, and the necessity of their documentation before any nutritional intervention. Therefore, the overall reduced endogenous antioxidant capacity of the CHA and SIM breeds compared to LIM might explain a possible failure in case a similar antioxidant treatment is implemented. On the contrary, the CHA breed revealed an increased CAT activity in the quadriceps, which was followed by lower protein carbonyl levels compared to the LIM breed. The observed tissue-specific results imply that distinct regulatory systems regulate the antioxidant defensive mechanisms.

The breed effects on lipid peroxidation are a critical endpoint that determine redox status equilibrium, and lipid and protein oxidation constitute the main causes of meat quality deterioration and the production of toxic compounds, affecting flavor and nutritional value [12,61]; hence, the search for possible associations of basal redox parameter levels in different tissues of beef cattle with meat quality is of interest. The study of meat quality is rather complex because it involves several aspects, both related to the product itself (microbiological profile, nutritional value) and other extrinsic factors (presentation, packaging, refrigeration and processing conditions). Moreover, the consumer perception of meat quality constitutes an imponderable aspect because it is mostly based on subjective experience traits (tenderness, flavor, color) [62]. In the present study, we used texture profile analysis and meat colorimetry to assess more objectively two of the main quality traits that define the consumer acceptance of beef meat. The results presented herein indicate that the GSH, TAC, CAT and CARBS levels affect the meat pH, color and texture parameters.

Specifically, the TAC levels in the blood were associated with higher values of meat color parameters (a*, b*, chroma, hue angle). Within the normal meat color ranges, high a* and chroma values combined with relatively low b* values are considered desirable because they represent a visually perceived vivid reddish color (a hue angle less than 40°) that consumers relate to fresh, quality beef meat. By contrast, a further increase of the hue angle is related to an unpreferable, darker, brownish color [38,63]. Because all of the ranges of the latter parameters were within the desirable limits in our study (Table 2), higher chroma and hue angle values lead to more reddish meat; hence, the blood TAC activity had a positive effect on the meat color. The latter is consistent with the fact that post-mortem oxidation processes in muscles lead to the formation of metmyoglobin, a chemical form of myoglobin that results in a brown meat color [63]. On the contrary, the liver TAC activity had a negative impact on a* and chroma, resulting in lower values. Moreover, the CAT and GSH activity in the muscle tissues (diaphragm, quadriceps) diversely affected meat color. More specifically, CAT in the diaphragm had a slightly increasing effect on b* and the hue angle, whereas GSH in the quadriceps had the opposite effect on b* and chroma. The relatively large β-coefficients and confidence intervals estimated for the effect of GSH are most likely due to the large scale differences among the involved variables (GSH range: 0.004–0.04 μmol/mg protein; b* range: 1.81–9.60; chroma range: 18.50–27.45) and the limited number of samples examined. The different regulatory mechanisms in the peripheral tissues and variations in the physiological functions and metabolic pathways that result in altered color biochemistry among different muscles [64], as well as the small population sample examined, might be the underlying causes of the contradictory results. Nevertheless, blood TAC may be more reliable as a biomarker of the overall antioxidant activity due to the central role of blood as the vital fluid of organisms, which is present in all tissues, including muscles. Hence, TAC in the blood may be an easy-to-obtain readout which might serve as an indicator of meat color. As such, blood TAC will allow, in the future, the ante-mortem non-invasive screening of animals to determine their cost with regard to meat quality, and possible options to improve it; this probability has already been proposed [65]. However, the present results need to be validated with a larger sample size. Furthermore, including meat samples of extreme color (i.e., discolored meat, cases of premature browning or persistent pinking) might give further insight into the sensitivity and specificity of TAC blood as a meat quality indicator.

Protein carbonylation has been found to adversely affect the meat color in other species (i.e., pigs), resulting in a pale and faded meat color [66]. In the present study, however, protein carbonyls in the liver and psoas major muscle were found to increase the hue angle and L* values, whereas protein carbonylation in the diaphragm decreased L*. The latter contradictory effects, which were very slight (β-coefficients: 0.015–0.959 by absolute value), might be due to the particularities of the present study: the small sample size and normal color values range (Table 2). In the study of Kazemi et al. [66], samples of distinctly different quality groups were examined; hence, the replication of the present study on a bigger sample with the inclusion of samples of different qualities might facilitate safer assumptions. Interestingly, we found no association of TBARS with meat color, whereas other studies have shown that lipid oxidation favors the formation of metmyoglobin, resulting in undesirable meat discoloration [67]. The latter odd finding might be due to the lack of extreme meat color values and the small sample size of the present study.

As one of the most important factors affecting meat color, pH was also studied. The impact of pH on meat color is both direct and indirect through the diverse metabolic pathways that regulate mitochondrial activity and protein oxidation, resulting in myoglobin oxidation and stability regulation [63,68]. In accordance with the fact that a post-mortem decreasing pH can favor protein oxidation in muscles [48], we found that protein carbonyls in the blood were associated with lower pH values, and the opposite was observed for increased GSH antioxidant activity in the psoas major muscle.

Antioxidant activity (CAT in the blood and psoas major, GSH in the quadriceps) adversely affected the meat tenderness (by increasing the hardness 1 and 2 and springiness values), whereas protein carbonylation (in the psoas major and diaphragm muscles) had the opposite effect (decreased hardness 1 and 2 and chewiness). The only exception to this pattern was the GSH activity in the blood, which was associated with decreased cohesiveness. The present results are in accordance with the recent findings of Malheiros et al. [69] based on a proteomics approach; tender meat was associated with muscle structural protein carbonylation and the increased oxidative stress of antioxidant proteins. The latter concept is further supported by earlier findings regarding meat aging; during the first week post-mortem, tender meat samples were associated with higher levels of proteolytic activity compared to tougher ones [70].

Based on the present results, the breed influences the basal levels of the redox parameters in blood and tissues of beef cattle. The variable levels of antioxidant activity, lipid and protein oxidation among the tissues indicate differences regarding the underlying regulatory mechanisms. The associations of the redox status with meat pH, color and tenderness show the potential for the use of the redox status biomarkers as indicators of meat quality. Among the studied tissues, blood is the most promising. Consequently, the characterization of the basal levels and the rectification of the redox parameters remain promising strategies to improve the quality of meat [71]. The regulation of these parameters is dependent on many ante- and post-mortem or genetic factors which have not yet been fully elucidated, representing a promising field of investigation that our study does not satisfy. This indicates that breed could also influence meat quality through muscle structure and physiology [50]. In response to the above, and to the fact that the LIM breed cattle exhibited decreased lipid peroxidation in our study and a previous one [51], it has also been proposed for the exhibition of higher scores on most of the meat quality characteristics such as lightness, redness and chroma [51], a phenomenon that necessitates investigation in our experiment as well. However, the present results need to be validated on a bigger sample. Furthermore, the inclusion of meat samples of different qualities will favor the interpretation of the relevant effects and clarify the specificity and sensitivity of different redox biomarkers as possible indicators of meat quality.

## 5. Conclusions

To the best of our knowledge, this is the first study to examine Greek beef meat quality parameters and redox potentials in tissues from animals that have not been submitted to experimental feed interventions. The redox biomarker baseline levels of different cattle breeds revealed the possible superiority of LIM cattle compared to CHA and SIM. Further studies on larger sample sizes are necessary to strengthen the validity of the present results. The differences in the antioxidant activity among the tissues indicated different underlying regulatory mechanisms and emphasized the need for further investigation to determine which are the most reliable as meat quality indicators. The associations of tissues’ redox status readouts with their meat quality parameters (pH, color, tenderness) indicate a potential for their use as indicators of meat quality. We propose that the determination of blood redox biomarkers can serve as important readouts that might further assist in the adoption of appropriate cattle management practices.

## Figures and Tables

**Table 1:** Redox biomarker baseline levels in the cattle tissues.

	*n*	Blood ^a^	Diaphragm ^b^	Liver ^b^	Psoas Major ^b^	Quadriceps ^b^
		Charolais				
GSH	13	6.4 (±0.76)	0.067 (±0.012)	0.063 (±0.042)	0.017 (±0.0075)	0.019 (±0.0067)
Catalase	13	48 (±9.8)	120 (±45)	7000 (±1200)	61 (±33)	48 (±19)
TBARS	13	7.2 (±1.3)	2.6 (±0.54)	3.9 (±0.81)	2.0 (±0.45)	1.8 (±0.41)
TAC	13	0.52 (±0.097)	0.29 (±0.054)	0.35 (±0.046)	0.31 (±0.035)	0.28 (±0.034)
Carbonyls	13	0.42 (±0.070)	4.0 (±2.6)	4.8 (±2.1)	3.1 (±1.3)	1.9 (±1.2)
		Limousin				
GSH	11	8.0 (±1.0)	0.084 (±0.020)	0.16 (±0.11)	0.040 (±0.010)	0.034 (±0.0084)
Catalase	11	54 (±5.3)	110 (±55)	6100 (±1400)	46 (±16)	29 (±12)
TBARS	11	6.4 (±0.95)	2.2 (±0.40)	2.9 (±0.67)	1.6 (±0.33)	1.5 (±0.36)
TAC	11	0.45 (±0.062)	0.34 (±0.066)	0.42 (±0.045)	0.33 (±0.044)	0.33 (±0.034)
Carbonyls	11	0.32 (±0.083)	2.3 (±1.9)	3.6 (±0.99)	3.9 (±1.5)	4.6 (±1.7)
		Simmental				
GSH	10	6.8 (±1.4)	0.060 (±0.010)	0.066 (±0.026)	0.031 (±0.011)	0.019 (±0.0078)
Catalase	10	55 (±16)	130 (±43)	4900 (±530)	73 (±45)	30 (±18)
TBARS	10	8.6 (±1.0)	2.2 (±0.36)	3.4 (±0.56)	2.3 (±0.54)	1.5 (±0.29)
TAC	10	0.73 (±0.078)	0.31 (±0.075)	0.36 (±0.064)	0.32 (±0.13)	0.33 (±0.080)
Carbonyls	10	0.38 (±0.068)	5.2 (±2.5)	4.5 (±1.1)	2.3 (±1.3)	4.1 (±1.7)

The data are represented as the mean ± SD. ^a^ Parameter concentration units in blood: GSH, μmol/g Hb; CAT, U/mg Hb; TBARS, μmol/L; TAC, mmol DPPH/L; CARBS, nmol/mg protein. ^b^ Parameter concentration units in diaphragm, liver, psoas major and quadriceps: GSH, μmol/mg protein; CAT, U/mg protein; TBARS, nmol/mg protein; TAC, mmol DPPH/mg protein; CARBS, nmol/mg protein.

**Table 2:** Descriptive statistics of the meat quality parameters.

	*n*	Mean	Standard Deviation	Median	Min	Max	Range	Skew	Kurtosis	Standard Error
L*	34	39.94	3.661	40.51	31.33	48.22	16.89	−0.28	−0.17	0.63
a*	34	22.98	1.854	23.12	18.41	25.82	7.41	−0.47	−0.34	0.32
b*	34	6.27	2.161	6.64	1.81	9.60	7.79	−0.26	−1.20	0.37
Chroma	34	23.88	2.219	23.77	18.50	27.45	8.95	−0.33	−0.42	0.38
Hue angle	34	0.26	0.076	0.29	0.10	0.38	0.28	−0.40	−1.21	0.01
pH	33	5.59	0.182	5.55	5.42	6.20	0.78	1.92	2.97	0.03
Hardness 1	33	1067.97	804.248	1022.23	225.79	3837.58	3611.79	2.16	5.02	140.00
Hardness 2	33	859.95	631.769	784.34	190.94	2976.72	2785.78	2.10	4.78	109.98
Springiness	33	0.69	0.103	0.68	0.46	0.95	0.48	0.09	−0.28	0.02
Cohesiveness	33	0.50	0.057	0.49	0.41	0.65	0.24	0.94	0.62	0.01
Chewiness	33	355.09	252.762	339.42	75.35	1172.40	1097.05	1.90	3.85	44.00

**Table 3:** Statistically significant effects of the redox status on the meat quality parameters (β-coefficients, *p*-value and 95% confidence intervals—CI).

Dependent Variable	Independent Variable	β-Coefficient	2.5% CI	97.5% CI	*p*-Value
Redness (a*)	TAC—blood	4.7128	0.8395	8.5861	0.0187
Redness (a*)	TAC—liver	−14.6856	−24.0230	−5.3483	0.0031
Yellowness (b*)	GSH—quadriceps	−65.6359	−127.3821	−3.8897	0.0380
Yellowness (b*)	TAC—blood	7.1183	2.9843	11.2524	0.0014
Yellowness (b*)	CAT—diaphragm	0.0186	0.0046	0.0325	0.0108
Lightness (L*)	CARBS—diaphragm	−0.5585	−0.9899	−0.1270	0.0129
Lightness (L*)	CARBS—psoas major	0.9592	0.1256	1.7929	0.0255
Chroma	GSH—quadriceps	−65.4472	−128.6614	−2.2330	0.0429
Chroma	TAC—blood	6.2854	1.8618	10.7090	0.0068
Chroma	TAC—liver	−16.6420	−27.7520	−5.5321	0.0046
Hue angle	TAC—blood	0.2388	0.0836	0.3940	0.0037
Hue angle	CAT—diaphragm	0.0007	0.0002	0.0012	0.0102
Hue angle	CARBS—liver	0.0153	0.0005	0.0301	0.0430
Log_pH	GSH—psoas major	1.0484	0.3271	1.7698	0.0058
Log_pH	CARBS—blood	−0.1299	−0.2568	−0.0031	0.0450
Log_hardness 2	CAT—psoas major	0.0067	0.0005	0.0129	0.0361
Log_hardness 2	CAT—blood	0.0195	0.0011	0.0378	0.0381
Log_hardness 2	CARBS—psoas major	−0.1584	−0.3113	−0.0055	0.0428
Log_hardness 1	CAT—psoas major	0.0070	0.0007	0.0134	0.0311
Log_hardness 1	CAT—blood	0.0207	0.0020	0.0394	0.0309
Log_hardness 1	CARBS—psoas major	−0.1622	−0.3189	−0.0055	0.0430
Springiness	GSH—quadriceps	5.8144	2.5477	9.0812	0.0011
Log_cohesiveness	GSH—blood	−0.0306	−0.0604	−0.0008	0.0443
Log_chewiness	CARBS—diaphragm	−0.0786	−0.1516	−0.0055	0.0359

## Data Availability

The data presented in this study are available on request from the corresponding author.

## Supplementary Materials

The following are available online at https://www.mdpi.com/article/10.3390/antiox10060958/s1; Table S1: Bulls ration composition.

## Abbreviations

LIM	Limousin cattle breed
CHA	Charolais cattle breed
SIM	Simmental cattle breed
GSH	reduced glutathione
CAT	catalase
TAC	total antioxidant activity
TBARS	thiobarbituric acid reactive substances
CARBs	protein carbonyls
