# Supplementary material for: Redox Biomarker Baseline Levels in Cattle Tissues and Their Relationships with Meat Quality
Source: Antioxidants (Basel). 2021 Jun 15;10(6):958. doi: 10.3390/antiox10060958 (PMC8232099; doi:10.3390/antiox10060958)
Supplement: Supplementary file 1 [file antioxidants-10-00958-s001.zip › antioxidants-1219157-supplementary.pdf]

Supplementary Table S1. Bulls ration composition

Diet composition					
60% of total ration	corn silage	65%			
	alfalfa hay	35%			
40% of total ration	corn grains	39%			
	wheat bran	12%			
	soybean meal	10%			
	barley grains	26%			
	ddgs	11%			
	minerals and vitamins premix	2%	Ca	19%	
			P	3,20%	
			Mg	2,80%	
			Na	8%	
			Vit A	520000	I.U./kg premix
			Vit D3	72000	I.U./kg premix
			Vit E	4000	mg/kg premix
			Zn	2400	mg/kg premix
			Mn	2400	mg/kg premix
			I	120	mg/kg premix
			Co	12	mg/kg premix
			Se	12	mg/kg premix
